# Is Exaggerated Release of Arginine Vasopressin an Endocrine Disorder? Pathophysiology and Treatment

**DOI:** 10.3390/jcm6110102

**Published:** 2017-10-31

**Authors:** San-e Ishikawa

**Affiliations:** Department of Endocrinology and Metabolism, International University of Health and Welfare Hospital, Nasushiobara 329-2763, Tochigi, Japan; saneiskw@jichi.ac.jp; Tel.: +81-287-39-3060

**Keywords:** impaired water excretion, hyponatremia, aquaporin 2, arterial underfilling, heart failure, SIADH, vasopressin (AVP) V_2_ receptor antagonist

## Abstract

Exaggerated release of arginine vasopressin (AVP) is profoundly involved in impaired water excretion and related hyponatremia. Such disorders underlie syndromes of inappropriate secretion of antidiuretic hormone (SIADH) and edematous diseases, such as congestive heart failure and decompensated liver cirrhosis. All the causes are fundamentally from non-endocrine diseases. AVP-induced water retention could produce hyponatremia, and further accelerate poor long-term outcome of edematous diseases. Administration of AVP V_2_ receptor antagonists verifies how much AVP is involved in the pathogenesis of the impaired water excretion. The present paper demonstrated that exaggerated release of AVP plays a crucial role as an accessory endocrine disorder in pathological states of water retention and dilutional hyponatremia in non-endocrine disorders.

## 1. Introduction

Endocrine disorders are fundamentally dependent upon the disorders of endocrine glands and their hormonal receptors. Regarding arginine vasopressin (AVP), three disorders are listed as endocrine diseases, namely, central and nephrogenic diabetes insipidus, and syndrome of inappropriate secretion of antidiuretic hormone (SIADH). Persistently inappropriate secretion of AVP produces impaired water excretion and hyponatremia in SIADH. However, there is no evidence of the disorders of endocrine glands in SIADH. SIADH include disorders of the central nervous system, intrathoracic disorders and drug administration, in addition to ectopic production of AVP in cancer tissues. Therefore, we do not know if SIADH is truly an endocrine disease. If we consider it from a different point of view, we can widen the category of disorders which are associated with exaggerated release of AVP. If so, we can include all disorders that have augmented release of AVP, even if their original diseases belong to non-endocrine organs.

Exaggerated release of AVP is profoundly linked to the development of water retention and edema in edematous diseases, including congestive heart failure (CHF) and decompensated liver cirrhosis [1,2]. Recently, it has been evident that AVP V_2_ receptor antagonists are effective in decelerating circulatory blood volume expansion and related dilutional hyponatremia in SIADH and CHF [3,4,5]. This could precede the category of exaggerated secretion of AVP in non-endocrine disorders as a sub-endocrinological disorder. In the present review, I note the pathophysiological state of excessive AVP secretion and possible treatments with AVP V_2_ receptor antagonists in various diseases.

## 2. Clinical Causes of Inappropriate Secretion of AVP

**Figure 1:**
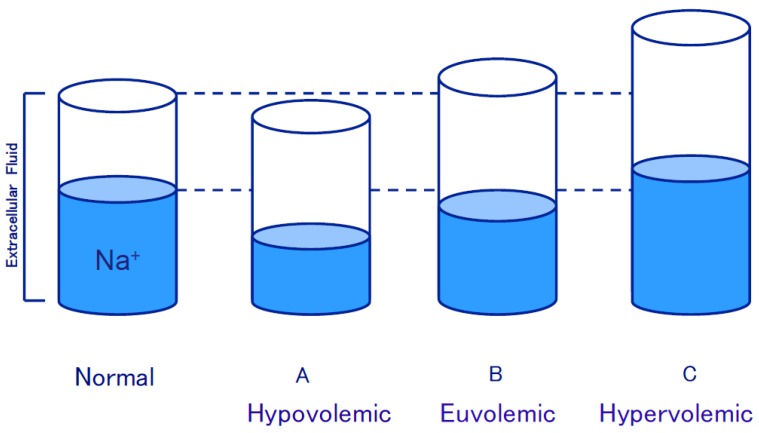
Three types of hyponatremia. (**A**) Hypovolemic hyponatremia; (**B**) Euvolemic hyponatremia; (**C**) Hypervolemic hyponatremia.

In some pathological states, release of AVP is persistently elevated although there is normal or increased circulatory blood volume. Such an augmented AVP release is “inappropriate”, thus providing impaired water excretion. Pathological states of water retention could develop dilutional hyponatremia, because extracellular sodium (Na) content remains unchanged, and the extracellular volume increases excessively. Namely, inappropriate secretion of AVP is in concert with pathological states of hyponatremia. As shown in Figure 1, there are three types of hyponatremia, depending on extracellular Na and extracellular volume. Hyponatremia accompanied by circulatory volume expansion (hypervolemic or dilutional hyponatremia, Figure 1C) is found in edematous diseases, including congestive heart failure, liver cirrhosis with ascites and nephrotic syndrome. The renin-angiotensin-aldosterone system and sympathetic nervous system, as well as AVP, are all mediated through arterial baroreceptors [1,6]. This mechanism is discussed in detail later. The second type is hyponatremia accompanyied by euvolemic state (euvolemic hyponatremia, Figure 1B). Such impairment in water excretion is found in SIADH, hypopituitarism, hypothyroidism and other disorders. The causes of SIADH are listed in Table 1. The diseases are not related to endocrine diseases or neurohypophyseal disorders.

In contrast, AVP is physiologically released under circulatory blood volume depletion. This is a homeostatic response, and the elevation of AVP release autonomically ceases after the circulatory blood volume returns to normal levels (Figure 1A).

## 3. Pathogenesis of Exaggerated AVP Release

### 3.1. Edematous Diseases

Renal Na and water excretion are predominantly regulated by the integrity of arterial circulation, that is they are determined by cardiac output and peripheral vascular resistance. Several baroreceptors on the high pressure side of the circulation can sense arterial underfilling. They are located in the carotid sinus, left atrium, aortic arch and renal afferent arterioles. Reduction of baroreceptor sensitivity could relieve the “tonic inhibition” on the afferent vagal nerve pathway of hormonal synthesis and secretion by the hypothalamo-neurohypophyseal system [7,8,9,10,11]. Such a reduction occurs when there is a reduction in systemic arterial pressure, cardiac stroke volume, renal perfusion or peripheral vascular resistance. In congestive heart failure, cardiac output is decreased following reduced stroke volume, though there is an increase in total circulatory blood volume. Low cardiac output could promote a decrease in “effective circulatory blood volume”, and impairs the sensitivity of baroreceptors. A reduction in effective circulatory blood volume is closely related to increases in the activity of the sympathetic nervous system, activation of the renin-angiotensin-aldosterone system and non-osmotic release of AVP. However, it remains unclear how baroreceptors sense the decrease in effective circulatory blood volume linked to low cardiac output in heart failure.

In decompensated liver cirrhosis, the pathogenesis of non-osmotic release of AVP is similar to that in heart failure. Peripheral arterial vasodilatation and arterio-venous fistula, primarily in the splanchinic vascular beds, have been proposed to account for the reduction in effective circulatory blood volume [12]. Such a phenomenon could reduce baroreceptor sensitivity, thus providing relief of tonic inhibition on hormonal secretion. Plasma AVP levels are persistently elevated with augmented expression of AVP mRNA in the hypothalamus in liver cirrhosis [13,14], and its elevation is further manifested in decompensated cirrhosis as compared to compensated cirrhosis. Furthermore, activation of the renin-angiotensin-aldosterone system and the sympathetic nervous system occur in cirrhosis, and the degree of activation is correlated with the progression of cirrhotic decompensation [15].

In edematous diseases, impaired water excretion is frequently linked to exaggeration of non-osmotic AVP release. The activation is mediated through baroreceptors, whose sensitivity is blunted under “arterial underfilling”. In addition, both the sympathetic nervous system and the renin-angiotensin-aldosterone system are activated, and increase Na reabsorption, followed by an increase in water retention. Because edematous diseases of heart failure and liver cirrhosis have excessive body water, these hormonal responses could be “inappropriate” and result in developing water retention.

### 3.2. SIADH

In patients with SIADH, plasma AVP is persistently elevated despite hypoosmolality. This is because AVP secretion is not suppressed inappropriately under hypotonic conditions, in which AVP release is reduced to undetectable levels in normal subjects [16]. There are two sources of AVP in SIADH: ectopic production of AVP in lung, pancreatic and other cancers, and increased central secretion of AVP from the hypothalamo-neurohypophyseal system due to central nervous system disorders, intrathoracic disorders or drugs. Normal values of plasma AVP are frequently observed in SIADH patients; these plasma AVP concentrations, however, are increased with respect to low plasma osmolality.

Ectopic production of AVP is clear, because AVP is ectopically synthesized in several cancer tissues and directly released into the systemic circulation. The produced AVP is biologically active. In contrast, there is no evidence regarding how afferent pathways transduce the stimulatory signals to AVP neurons in hypothalamus in central nervous system and lung disorders. In part, a possible mediator maybe vasospasm of central brain arteries in the disorders of subarachnoid hemorrhage. Disruption of extracranial vascular aneurysms may be involved in triggering AVP release from the hypothalamo-neurohypophyseal system [17]. However, the signal and its pathways are still unknown.

Hypothalamic AVP neurons are passively activated through stimulatory signals from the disorders of non-endocrine organs and tissues, thus providing an increase in endogenous AVP release in SIADH. This enhanced AVP release is a time-limited phenomenon, except for ectopic production of AVP.

## 4. Impaired Water Excretion

Several studies in animal models of impaired water excretion have shown that enhanced hydro-osmotic action of AVP depends on non-suppressible release of AVP. In an experimental model of SIADH in rats, serum Na levels decreased to below 120 mmol/L within 24 h, and hyponatremia persisted during a 14-day observation period. This animal model was made by the subcutaneous administration of the AVP V_2_ agonist, 1-deamino-8-D-arginine vasopressin (DDAVP), by an osmotic mini pump, and offering a liquid diet [18]. Aquaporin 2 (AQP2) is an AVP-dependent water channel of collecting duct cells [19]. Receptor occupancy with AVP in the renal collecting duct allows the activation of adenylate cyclase to produce cAMP. cAMP activates cAMP-dependent protein kinase A (PKA), and phosphorylation of PKA then mediates cellular signaling of AVP to the AQP2 water channel. This leads to translocation of AQP2 from membranes of cytoplasmic vesicles to the apical plasma membrane (short-term regulation), and increases AQP2 transcription and protein synthesis (long-term regulation). The expression of AQP2 mRNA was upregulated throughout the 14-day observation period. The peak of mRNA expression occurred at day 2, and was followed by a gradual decrease thereafter [20,21]. The increase in AQP2 mRNA expression was reversed three hours after oral administration of the non-peptide AVP V_2_ receptor antagonist OPC-31260. The mRNA results were paralleled by changes in AQP2 protein expression [20,21].

An experimental rat model of liver cirrhosis with ascites was made by the subcutaneous administration of carbon tetrachloride (CCl_4_) for three months. AQP2 mRNA and protein expression were increased in these cirrhotic rats [20]. An acute water load clarified the impairment in renal water excretion, a finding associated with the non-suppressible release of AVP. The administration of OPC-31260 totally reversed the increase in AQP2 mRNA expression.

Increases in AQP2 mRNA expression and protein abundance were found in experimental models of congestive heart failure in rats. Xu et al. [22] stimulated chronic heart failure by ligating the descending limb of the left coronary artery. Cardiac output and plasma osmolality were significantly decreased, and plasma AVP increased in the chronic heart failure rat models compared to the sham-operated rats. AQP2 mRNA and protein abundance were both significantly increased in the kidneys of chronic heart failure rats. Similarly, Nielsen et al. [23] showed enhanced trafficking of AQP2 to the apical plasma membrane in collecting ducts of rats with congestive heart failure. Administration of OPC-31260 significantly reduced AQP2 mRNA expression and protein abundance.

**Figure 2:**
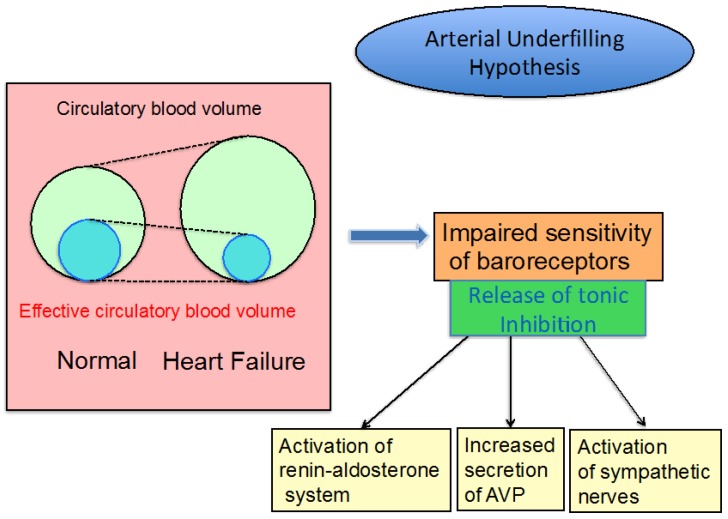
Hypothesis for dysregulation of baroreceptor-mediated hormonal release in congestive heart failure.

In edematous diseases of congestive heart failure and liver cirrhosis with ascites, exaggerated release of AVP is persistent under arterial underfilling [1,2,7]. Continuous baroreceptor-mediated AVP release further impairs renal water excretion in the state of water retention, contributing to acceleration of edematous change. Also, the activation of the renin-angiotensin-aldosterone and sympathetic nervous systems produce renal Na reabsorption, thus providing water retention secondarily (Figure 2). These synergistic changes of baroreceptor-mediated hormonal activation under arterial underfilling, which reduce renal water reabsorption, may not take place in edematous diseases. Taken together, the hypervolemic state is pathologically maintained throughout edematous diseases. 

In contrast, circulatory blood volume is different in SIADH. Initially, elevation of plasma AVP levels increase renal water reabsorption and produce a hypervolemic state. Thereafter, the body moves gradually to an euvolemic steady state. This change is derived from “AVP escape”, and an increase in renal Na excretion. Sustained elevation of AVP release produces downregulation of receptor binding capacity of AVP V_2_ receptors in the collecting duct, but cellular signaling of AVP remains augmented. In response to this signal, expression of AQP2 mRNA and protein abundance are increased, and produce sustained elevation of water reabsorption. Prompt increase in extracellular fluid dilutes it because of no change in Na content, and there is a shift to a hypotonic condition, which partially diminishes the augmented expression of AQP2 mRNA and protein abundance as described earlier in the animal model of SIADH [21,24]. Therefore, maximal urinary concentration is partially decelerated, followed by an increase in urine volume. In the steady state extracellular volume gradually decreases toward a normal volume, moving away from the initial volume expansion.

In SIADH, Na metabolism also contributes to decrease circulatory blood volume. Increased circulatory blood volume allows an increment in renal blood flow, which modulates renal Na handling. These results in enhanced glomerular filtration, inhibition of the renin-angiotensin-aldosterone system, augmented secretion of natriuretic peptide, and increases in renal prostaglandin synthesis and the kinin-kallikrein system. These alterations all enhance renal Na excretion, resulting in an increase in renal water excretion. The compensation in water and Na metabolism contribute to reduce the increased circulatory blood volume, and produce a new steady “euvolemic” state. Such alterative changes do not occur in edematous diseases.

**Figure 3:**
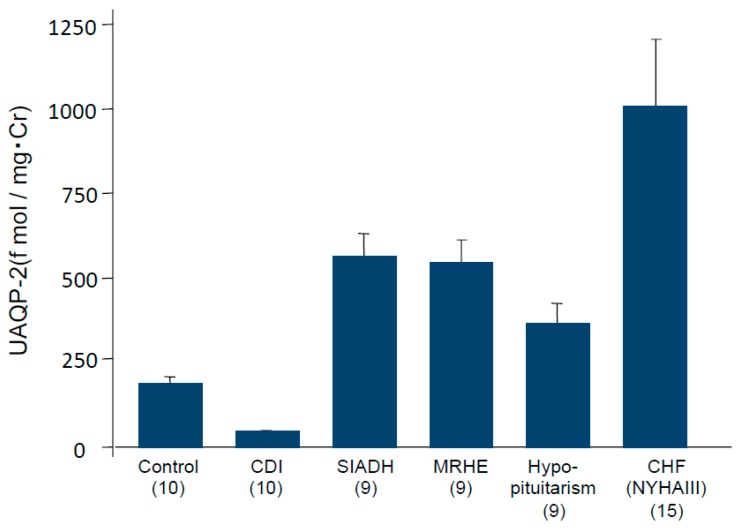
Elevation of urinary excretion of aquaporin 2 (UAQP2) in patients with impaired water excretion. The numbers of patients are shown in brackets.

Urinary excretion of AQP2 (UAQP2) was detected in both soluble and membrane-bound forms by Western blot analysis [25]. Immunoblots of urine samples showed AQP2 with molecular sizes of 29 kDa and 40–50 kDa. The band at 40–50 kDa represents a glycosylated form of the 29 kDa protein. UAQP2 can be determined quantitatively by radioimmunoassays or ELISAs [26,27]. The fraction of AQP2 excreted into the urine is approximately 3% of the AQP2 present in renal collecting duct cells [28]. We found positive correlation of UAQP2 with plasma AVP levels in normal subjects [26]. As shown in Figure 3, UAQP2 was markedly elevated in patients with SIADH, hypopituitarism, mineralocorticoid-responsive hyponatremia of the elderly (MRHE) and congestive heart failure [29,30]. In these disorders, plasma AVP levels were persistently increased. In contrast, UAQP2 was depressed in central diabetes insipidus, where AVP secretion is absent. Furthermore, we demonstrated that there was also a positive correlation between UAQP2 and plasma AVP levels in congestive heart failure [30]. Taken together, these findings suggest that elevation of UAQP2 is derived from the exaggerated hydroosmotic action of AVP in a pathological state of impaired water excretion. Measurement of UAQP2 is a useful tool for evaluating water metabolism disorders.

## 5. Hyponatremia Predicts Poor Prognosis

Hyponatremia is relatively common in congestive heart failure. Gheorghiade et al. [31] reported that hyponatremia of less than 135 mmol/L was found in 19.7% of 48,612 patients. There are several reports regarding the relation of hyponatremia with prognosis in congestive heart failure. In the short-term, length of stay in hospital and in-hospital mortality were greater in patients having less than 135 mmol/L hyponatremia than those with hyponatremia greater than 135 mmol/L [31]. We reported that hyponatremia predicts long-term prognosis in heart failure patients receiving cardiac resynchronization therapy (CRT) [32]. Seventy-seven patients who were all in NYHA II, III or IV classes, and their left ventricular ejection fraction on echocardiogram was less than 35%, were examined. During a mean follow-up period of 601 days, 22 of 77 patients (29%) had a cardiovascular event. In multiple analysis, hyponatremia was the only independent factor associated with the occurrence of heart failure rehospitalization and cardiac death (Hazard ratio 0.82, *p* = 0.034). Similar results were obtained in other studies [33,34]. Low serum Na levels at admission were closely associated with increased post-discharge mortality and rehospitalization in patients with heart failure. Dilutional hyponatremia is a consequence of persistent AVP-induced water retention due to “arterial underfilling”, as described earlier. Simultaneous circulatory blood volume expansion, in association with hyponatremia, could increase cardiac preload, thus accelerating dysfunction of cardiac contraction in heart failure (vicious cycle) [7,35].

Similarly, hyponatremia is found in decompensated cirrhosis [12,13]. As mentioned above, baroreceptor-mediated hormonal activation promotes water and Na retention in cirrhosis with ascites under arterial underfilling. Post-discharge mortality is increased when hyponatremia becomes manifested [36,37,38]. Several reports found that survival rates progressively decreased to below 40% within 3–6 months if serum Na levels were 130 mmol/L or less. Thus, occurrence of hyponatremia reflects serious condition in cirrhotic patients with ascites. This hypervolemic hyponatremia is also based on an inappropriate increase in body fluid.

Euvolemic hyponatremia is a hallmark of SIADH. There is no report that hyponatremia may affect prognosis in SIADH in the literature.

## 6. AVP V_2_ Receptor Antagonists and Impaired Water Excretion

As aforementioned, we have demonstrated that exaggerated release of AVP results in impaired water excretion. Therapeutic approaches for blocking the antidiuretic action could ameliorate an inappropriate increase in body fluid. Now, such a therapy is possible, because non-peptide AVP V_2_ receptor antagonists can be clinically available to patients [39,40].

We observed the diuretic effects of AVP V_2_ receptor antagonist in experimental SIADH rats [41]. The SIADH rat model was made by the subcutaneous administration of the V_2_ agonist, DDAVP, by osmotic minipumps, and offering a liquid diet. Serum Na levels were decreased to below 120 mmol/L within 24 h, and hyponatremia was maintained in association with a concentrated urine throughout the 14-day observation period. The oral administration of the non-peptide AVP V_2_ receptor antagonist OPC-31260 started on day 7, and continued once a day for the duration of the experiment. This maneuver promptly normalized serum Na levels in 12 h, in association with an increase in urine volume and a decrease in urinary osmolality. The normalization of serum the Na level was maintained during the rest of the experimental period.

The administration of carbon tetrachloride (CCl_4_) for three months produced liver cirrhosis with ascites in rats. Non-suppressible release of AVP was obtained in the CCl_4_-induced cirrhotic rats. An acute water load (30 mL/kg) verified a reduction in percent changes of excreted water, and an increase in urinary osmolality in cirrhotic rats as compared to normal rats. Such impaired water excretion was totally improved by orally administering OPC-31260 [42]. These in vivo experiments demonstrate that the impaired water excretion associated with non-suppressible AVP release can be reversed.

We have previously demonstrated the efficacy of mozavaptan (OPC-31260) in hyponatremia in patients with SIADH [43]. A single intravenous injection of mozavaptan increased urine volume and decreased urinary osmolality, providing a 3 mmol/L increase in serum Na levels during the 4-h observation period. Schrier et al. [3] reported study of ascending levels of Tolvaptan in Hyponatremia (SALT)-1 and SALT-2 studies in the United States and Europe. They studied the efficacy of tolvaptan in hyponatremic patients, including SIADH, liver cirrhosis and congestive heart failure. In the tolvaptan group, serum Na levels were 128.5 ± 4.5 mmol/L. After administering tolvaptan, serum Na levels normalized during the 30-days observation period. Serum Na levels increased by 3.7 ± 2.7 mmol/L at four days, and by 6.2 ± 4.1 mmol/L at 30 days. In contrast, serum Na levels remained unchanged during the 30-day observation period in the placebo group. The efficacy of tolvaptan was mostly observed in the subgroup of SIADH patients. Berl et al. [44] observed 111 patients who were part the SALT study. After completing the SALT study, these 111 patients had been treated with tolvaptan for an additional four years. Serum Na levels were continuously kept within the normal range during the four-year observation period (SALTWATER study).

Gheorghiade et al. [45] demonstrated that tolvaptan increased diuresis in patients with congestive heart failure. Oral administration of tolvaptan increased urine volume, reduced body weight and increased serum Na levels in heart failure patients with NYHA classes I and II. Similar results were also obtained in advanced heart failure patients with NYHA classes III and IV [4]. Konstam and his associates [5] studied 4133 patients with congestive heart failure in 359 institutes in the United States (EVEREST study). Then mean follow-up period was 9.9 months. In the short-term, clinical symptoms of dyspnea, orthopnea, fatigue and edema were markedly improved in the tolvaptan group compared to the placebo group [46]. The patients who had greater reduction in body weight also had greater water diuresis, improved clinical symptoms and shortened in-hospitalization. In the long-term, there was a significantly greater decrease in body weight and increase in serum Na levels in the tolvaptan group compared to the placebo group. However, Kaplan-Meyer analysis observed no difference in cardiac death and rehospitalization of heart failure between the tolvaptan and the placebo groups [5].

In Japan, tolvaptan has been clinically available for treating congestive heart failure [47]. Tolvaptan is effective for increasing water diuresis, thus resulting in rapid reduction of congestion. However, clinical experience has clarified that approximately 20–40% of patients fail to respond to tolvaptan, and that they had little alteration in urine volume and urinary osmolality [48,49]. We now recognize that there are two groups: responders and the non-responders. At the present time, the mechanism that underlies the unresponsiveness to tolvaptan is not understood. It has been about six years since tolvaptan has been used for treating heart failure in Japan. There are a few reports evaluating the long-term outcome of heart failure. Uemura et al. [50] documented that tolvaptan significantly reduced cardiac mortality and re-hospitalization of heart failure patients, but did not modify the survival rate in 102 patients with congestive heart failure. On the other hand, Matsue et al. [51] reported that there was no change in the long-term outcome after tolvaptan treatment in 217 patients with acute heart failure. Their additional analysis revealed that tolvaptan improved combined events of heart failure re-hospitalization and all-cause death, in patients with eGFR of 30 mL/min/1.73 m^2^ or above. Further studies will be necessary to exactly determine whether tolvaptan affects long-term outcomes in patients with heart failure.

The aforementioned SALT study included cirrhotic patients who accounted for one third of the hyponatremic patients [3]. Serum Na levels significantly increased in association with water diuresis. Several groups reported that tolvaptan increased serum Na levels in decompensated cirrhotic patients [52,53]. A clinical trial study was performed in 164 cirrhotic patients with ascites in Japan [54]. The tolvaptan group (82 patients) increased water diuresis and serum Na levels by about 2–6 mmol/L, compared to the placebo group which had no change in water diuresis.

## 7. Conclusions

In this review, I have demonstrated that exaggerated release of AVP is profoundly involved in impaired water excretion in edematous diseases, as well as in SIADH. All the causes are fundamentally from non-endocrine disorders. Administration of AVP V_2_ receptor antagonists provides evidence that AVP directly enhances water retention, and demonstrates AVP involvement in the pathogenesis of impaired water excretion. These findings indicate that exaggerated release of AVP plays a crucial role as an accessory endocrine disorder in pathological states of water retention and related dilutional hyponatremia in non-endocrine disorders.

## Figures and Tables

**Table 1:** Causes of SIADH.

Cancer	Diseases of Central Nervous System	Intrathoracic Diseases	Drugs
Lung cancer	Encephalils	Pneumonia	Vincristine
Duodenal cancer	Meningitis	Tuberculosis	Cyclophosphamide
Pancreatic cancer	Cerebrovascular diseases (Cerebral infarction and hemorrhage)	Pulmonary abscess	Clofiblate
Malignant lymphoma	Subarachnoid hemorrhage	Pulmonary fungus diseases	Carbamazepine
Prostatic cancer	Subdural hematoma	Lung cancer (non-ectopic production)	Nicotine
Ewing sarcoma	Head injury		
	Acute porphyria		
	Pituitary tumor		
